# Ontology-based approach for *in vivo* human connectomics: the medial Brodmann area 6 case study

**DOI:** 10.3389/fninf.2015.00009

**Published:** 2015-04-10

**Authors:** Tristan Moreau, Bernard Gibaud

**Affiliations:** Medicis, UMR 1099 LTSI, INSERM, University of Rennes 1Rennes, France

**Keywords:** ontology, connectome, data sharing, neuroanatomy, neuroimaging, tractography, semantic web, MRI

## Abstract

Different non-invasive neuroimaging modalities and multi-level analysis of human connectomics datasets yield a great amount of heterogeneous data which are hard to integrate into an unified representation. Biomedical ontologies can provide a suitable integrative framework for domain knowledge as well as a tool to facilitate information retrieval, data sharing and data comparisons across scales, modalities and species. Especially, it is urgently needed to fill the gap between neurobiology and *in vivo* human connectomics in order to better take into account the reality highlighted in Magnetic Resonance Imaging (MRI) and relate it to existing brain knowledge. The aim of this study was to create a neuroanatomical ontology, called “Human Connectomics Ontology” (HCO), in order to represent macroscopic gray matter regions connected with fiber bundles assessed by diffusion tractography and to annotate MRI connectomics datasets acquired in the living human brain. First a neuroanatomical “view” called NEURO-DL-FMA was extracted from the reference ontology Foundational Model of Anatomy (FMA) in order to construct a gross anatomy ontology of the brain. HCO extends NEURO-DL-FMA by introducing entities (such as “MR_Node” and “MR_Route”) and object properties (such as “tracto_connects”) pertaining to MR connectivity. The Web Ontology Language Description Logics (OWL DL) formalism was used in order to enable reasoning with common reasoning engines. Moreover, an experimental work was achieved in order to demonstrate how the HCO could be effectively used to address complex queries concerning *in vivo* MRI connectomics datasets. Indeed, neuroimaging datasets of five healthy subjects were annotated with terms of the HCO and a multi-level analysis of the connectivity patterns assessed by diffusion tractography of the right medial Brodmann Area 6 was achieved using a set of queries. This approach can facilitate comparison of data across scales, modalities and species.

## 1. Introduction

The human brain is constituted of a vast amount of interconnected neurons forming structural circuits which transmit information. Multi-scale analysis of this *anatomical connectivity* (Caspers et al., 2013) from synaptic connections between individual neurons (microscopic scale), to brain regions interconnected via white matter fiber bundles (macroscopic scale) is fundamental to better apprehend the link between structure and function in diseased and healthy brains (Honey et al., 2010). A promising way for studying brain connectivity is to compile a coherent mapping of the network of elements and connections forming the human brain and defined as the *human connectome* (Sporns et al., 2005).

Recent advances in Magnetic Resonance Imaging (MRI) and brain networks have opened new possibilities to map and analyse anatomical and functional long-range connectivities in the living brain, giving birth to a new field of research: *human connectomics* (Behrens and Sporns, 2012). Currently, diffusion MRI (dMRI) and functional MRI (fMRI) are the most popular modalities to assess non invasively anatomical and functional connectivities, respectively (Craddock et al., 2013). dMRI estimates the local fiber bundles orientations at millimeter voxel resolution as the directions of least hindrance to water diffusion in brain. Then, tractography aims at reconstructing white matter fiber bundles using algorithmic approaches based on local fiber bundles orientations (Basser et al., 2000). fMRI uses temporal correlations in the fluctuations of the Blood-Oxygenation-Level-Dependent (BOLD) signal to infer functional connectivity (Smith et al., 2011). After reconstruction of anatomical or functional connectivities from MRI (Jbabdi and Johansen-Berg, 2011), *in vivo* neuroimaging data can be modeled and analyzed using connectomics in order to produce brain networks at macroscopic scale (~ 1 cm^3^ or greater) (Hagmann et al., 2007; Zalesky et al., 2011; Sporns, 2013). However, there is a great diversity in methodological approaches, especially no consensus currently exists on how to best define *nodes* for charting *in vivo* human connectome, i.e., subdividing the brain into macroscopic regions in an anatomo-functional coherent way (Craddock et al., 2013; Fortino et al., 2013). Indeed, depending on the scope of the study, nodes can represent small regions (~ 1 cm) or larger brain areas as a specific gyrus. Moreover comparing data across scales, modalities, and species remains challenging (Essen and Ugurbil, 2012; Leergaard et al., 2012). A real need exists of new neuroinformatics tools for *in vivo* human connectomics that allow different levels of granularity of multi-modal connectivity data to be described, shared, integrated and compared.

Semantic annotation of brain images consists in associating meaningful metadata using terms of an ontology in order to describe and share information related to that resource such as acquisition protocol, anatomical content, diagnosis etc. (Mechouche et al., 2009; Turner et al., 2010). Biomedical ontologies are structured vocabularies representing classes of entities which are of biomedical significance in reality. They focus on the definition of the entities of the domain being modeled and on the relations between them, especially the subtype relation used to organize the entities in a taxonomy. Ontologies also specify other relations, such as the “part of” relation, or any other relation that is relevant in the domain of interest. Specifying the set of relations (called axioms) that apply to all the instances of a class contributes to capture knowledge about this entity (Gruber, 1995). Axioms can be expressed in the OWL^1^ ontology language standard (Web Ontology Language, defined by the W3C), and especially the OWL DL^2^ sublanguage, based on Description Logics (DL) (Baader et al., 2003). OWL DL provides a good compromise between expressivity and computational complexity (and decidability). Moreover, it allows reasoning on formal knowledge and infering automatically new axioms using description logic reasoning engines such as FaCT++^3^. Ontology-based systems and reasoning engines are particularly relevant in the human connectomics realm as they provide the capability to apprehend consistently multi-scale knowledge, to describe heterogeneous data with semantic annotations, and finally to facilitate data querying, sharing and interoperability.

In recent years, different efforts were reported to specify computer models and ontologies to represent, collate, process and share human brain anatomical connectivity (OBO Relation Ontology, Smith et al., 2005; Swanson and Bota, 2010; Larson and Martone, 2013; Bota et al., 2014; Nichols et al., 2014). On one hand, the Foundational Model of Anatomy (FMA) was developed to provide a reference ontology for human anatomy. It includes many terms from Terminologia Anatomica (Federative Committee on Anatomical Terminology, 1998), which itself founds its origin in Nomina Anatomica (International Anatomical Nomenclature Committee, 1989). The foundamental difference between these terminologies and ontologies like FMA is that the former provide organizations of terms that enhance part of the intrinsic meaning of each term, in an implicit way, whereas ontologies such as FMA relate terms using relationships bearing explicit semantics such as subsumption links and “part of” links. FMA specifies anatomical connectivity relationships at different levels of granularity (Rosse and Mejino, 2003; Nichols et al., 2014). On the other hand, the Foundational Model of Connectivity (FMC) provides a high level conceptual framework suitable for modeling “structural architecture of nervous connectivity in all animals at all resolutions” (Swanson and Bota, 2010). In particular, this model influenced and is compatible with BAMS, the Brain Architecture Management System built by Mihail Bota and co., a neuroinformatics system to store, mine and model structural connectivity in multiple species such as mouse, rat, cat, macaque and human. Most connectivity data concern pathway-tracing experiments in animals, techniques based on injection of a tracer and tracing of neural connections either from their source to their point of termination (anterograde tracing) or the opposite (retrograde tracing). However, although these biological ontologies and conceptual models aim at representing anatomical connectivity, none of them can be used to represent connectivity assessed by diffusion tractography, yet. Indeed, diffusion tractography can only provide limited insight on the organization of *in vivo* white matter fiber bundles at the present time (cf. Section 4): for example it cannot determine polarity of connections, nor synaptic connections. Moreover, cytoarchitecture of the cerebral cortex cannot be rendered using MRI due to limited spatial resolution, so that concepts of gray matter region defined using criteria based on spatial distribution of a set of neuron types are not relevant for *in vivo* connectomics. So, a real need is emerging of new ontology in order to bridge the gap between experimental neurobiology and *in vivo* human connectomics observations provided by MRI.

An important and complementary field of research in neuroimaging concerns the development of digital atlases providing both a template brain and neuroanatomical labels in a conformed space. Individual brain datasets are aligned to the atlas using volumetric or surface-based registration approaches in order to propagate the neuroanatomical labels of the atlas to brain regions. A great number of *in vivo* neuroimaging datasets are currently annotated using brain atlases such as the Talairach Atlas (Talairach and Tournoux, 1988), the Montreal Neurological Institute (MNI) atlas (Tzourio-Mazoyer et al., 2002), or the atlases embedded in software tools such as Freesurfer^4^ (Fischl et al., 2004; Desikan et al., 2006) or the JHU white matter tractography atlas^5^ (Wakana et al., 2007; Hua et al., 2008). Recently, FMA provided a mapping between several terminologies used in brain atlases, such as Freesurfer or the JHU white matter tractography atlas, and the corresponding neuroanatomical concepts defined in FMA (Nichols et al., 2014). This effort facilitates the use of FMA as a reference and pivotal terminology for the annotation of brain segmentation results such as cortical or subcortical gray matter regions, and different white matter fiber bundles.

The main contribution of this paper was to create a generic neuroanatomical ontology called “Human Connectomics Ontology”^6^ (HCO) in order to represent macroscopic regions defined on MRI datasets connected via fiber bundles assessed by diffusion tractography in the living human brain. Grounded on the FMA reference ontology, HCO was expressed in OWL and used the OWL DL sublanguage in order to be processable by usual reasoning engines. The latter provide highly optimized implementations of reasoning algorithms to process and correctly answer arbitrarily complex queries, such as those involving, e.g., transitive part-whole and spatial relationships. Moreover, an experimental work was achieved in order to show how the HCO could be effectively used to address complex queries concerning *in vivo* MRI connectomics datasets: a multi-level analysis of the connectivity pattern of the right medial Brodmann Area 6 (BA6) reconstructed by diffusion tractography was achieved, using a set of queries on annotated neuroimaging datasets of five healthy subjects. The medial BA6 region is a cortical region defined using a set of cytoarchitectural criteria (Zilles and Amunts, 2010) and is part of the medial frontal cortex located on the midline surface of the hemisphere just in front of the primary motor cortex. This region of interest was chosen because different studies showed how it could be subdivided into different sub-regions in a reproducible way using criteria based on long-range connectivity assessed by diffusion tractography (Johansen-Berg et al., 2004; Anwander et al., 2007; Jbabdi et al., 2009). We believe that this approach can facilitate comparison of data across scales, modalities and species.

The following of the paper is organized as follows. Section 2 describes how the HCO was designed and achieved. Section 3 is related to the experimental work. Finally, Sections 4 and 5 are dedicated to the discussion and conclusion.

## 2. Materials and methods

### 2.1. A neuroanatomical ontology for *in vivo* human connectomics

#### 2.1.1. Requirements and design

Competency questions (Neuhaus and Vizedom, 2013) are more and more used to specify the domain that an ontology should cover. Therefore, we designed a set of competency questions pertaining to a use case inspired by the medial BA6 connectivity-based parcellation (Johansen-Berg et al., 2004), in order to assess how the Human Connectomics Ontology (HCO) can support hypothesis-driven analysis of connectomics datasets at different levels of granularity:

- Which gray matter parts of the right superior frontal gyrus have a connectivity pattern passing through the corticospinal tract or through some gray matter parts of the right precentral gyrus?
- Which gray matter parts of the right superior frontal gyrus have a connectivity pattern passing through some gray matter parts of the right medial parietal cortex or through some gray matter parts of the right inferior frontal cortex?
- Which gray matter parts of the right superior frontal gyrus have a connectivity pattern passing through the corticospinal tract or through some gray matter parts of the right precentral gyrus or through some gray matter parts contiguous with the right precentral gyrus?
- Which anatomical white matter fiber bundles connect some gray matter parts of the right superior frontal gyrus to some gray matter parts of the right temporal lobe?

In order to meet these requirements, a neuroanatomical ontology module, called “NEURO-DL-FMA,” was first constituted in order to annotate gross anatomy of the brain (i.e., gray matter regions, white matter fiber bundles). NEURO-DL-FMA was based on a subset of FMA which is an open source reference ontology representing the phenotypic structure of the human body at different scales. FMA contains more than 85,000 classes and 140 relationships between entities (Rosse and Mejino, 2003; Golbreich et al., 2013). Finally, the HCO was based on NEURO-DL-FMA and aimed at representing nodes connected with fiber bundles assessed by diffusion tractography. Moreover, nearest neighbor topology between gray matter regions was also represented.

**Figure 1:**
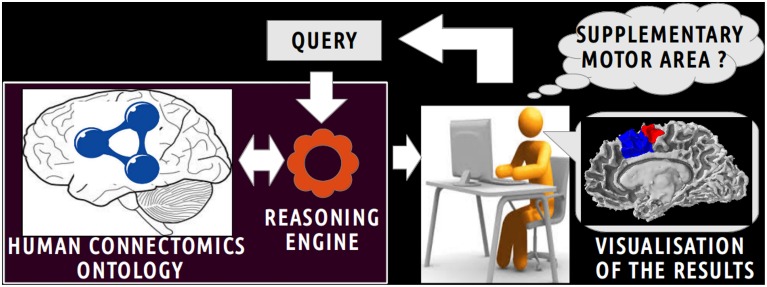
**Scenario of information retrieval using the Human Connectomics Ontology (HCO).** On the central part of the figure, an investigator can pose a wide range of queries using terms of the HCO: as an illustration, it could be to retrieve all cortical parcels belonging to the supplementary motor area. On the left part of the figure, the query is submitted to a reasoning engine that infers automatically part-whole, connectivity and spatial relationships at different level of granularity. On the right side of the figure, the results of the query can be easily visualized.

Figure 1 depicts a scenario of information retrieval concerning *in vivo* connectivity patterns assessed by diffusion tractography. Investigators can pose a wide range of queries using terms (i.e., classes and object properties) of the HCO. For example, an investigator could be interested in retrieving all cortical parcels of the right medial BA6 which have a connectivity pattern similar to the right Supplementary Motor Area (connectivity pattern passing through the right corticospinal tract or connected to gray matter parts of the right precentral gyrus). The query is submitted to a reasoning engine that infers automatically part-whole, connectivity and spatial relationships at different levels of granularity.

#### 2.1.2. NEURO-DL-FMA: a neuroanatomical gross anatomy ontology

The NEURO-DL-FMA is a neuroanatomical gross anatomy ontology that was achieved in two steps. First all useful entities and relations were extracted as a “view” from the FMA reference ontology (OWL Full 3.2.1 version) (Noy and Rubin, 2008). This view was then translated into OWL DL, which was necessary since most commonly used reasoning engines do not support OWL Full.

As the FMA contains more than 85,000 anatomical concepts, the first step was to extract a “view” from the FMA in order to focus only on concepts and relationships of interest. This was achieved using vSparQL queries (Shaw et al., 2011) and the entities were extracted from the more specific to the more general ones. This view was constituted of (1) all concepts denoting a gray matter structure mapping a Freesurfer cortical region, (2) all concepts denoting a white matter bundle mapping an entity in the JHU white matter tractography atlas. A one-to-one mapping between FMA and the Freesurfer and JHU white matter tractography atlas terminologies was available in the 3.2.1 version of the FMA (Nichols et al., 2014). Then all the entities related using the *fma:regional_part_of* or *fma:constitutional_part_of* object properties were extracted recursively (the “fma” prefix denotes terms originating from the FMA). The Brodmann areas, the hippocampus parts, and the other object and data properties were excluded. All the entities that subsume the entities present in the current view were included recursively. All the meta-classes of FMA (Dameron et al., 2005) included in our view were then discarded as they did not contain useful information for our gross anatomy ontology. All concepts that did not concern the domain of neuroanatomical gross anatomy such as *fma:Human_body* were also discarded. In order to reuse nearest neighbor topology knowledge represented in the FMA, all the entities related using the *fma:attributed_continuous_with* object property of type *fma:Continuous_with_relation* were included in our view. Finally, only the following three object properties and their inverse (if exists) were kept: *fma:constitutional_part*, *fma:regional_part*, *fma:attributed_continuous_with*.

**Figure 2:**
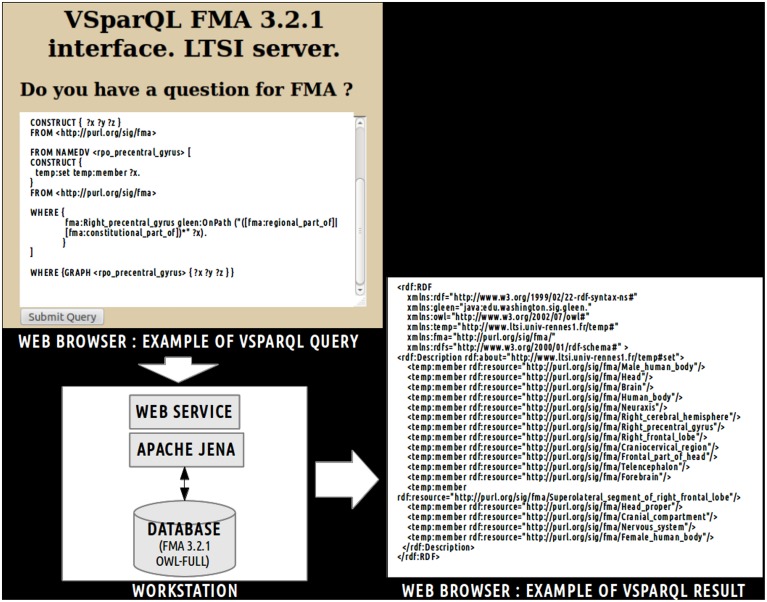
**Example of vSparQL query submitted to a web service based on Apache Jena that permitted to extract portions of the Foundational Model of Anatomy (FMA) embedded in a MySQL database by recursively following complex pathways within the ontology graph.** On the superior part of the figure, a vSparQL query aimed to extract all anatomical entities related to the *fma:Right_precentral_gyrus* (the “fma” prefix denotes that the entity was part of the FMA) following the *fma:regional_part_of* or *fma:constitutional_part_of* object properties. The right part of the figure shows the result of the query, expressed in RDF (Resource Description Framework).

This view was achieved using a web service implementation developed by the University of Washington's Structural Informatics Group^7^. In this implementation, the FMA (OWL Full 3.2.1 version) is embedded in a *MySQL*^8^ relational database. This web service based on *Apache Jena*^9^ accepts *VSparQL* queries allowing portions of the FMA to be extracted by recursively following complex pathways within the ontology graph (Shaw et al., 2011). Figure 2 shows an example in which all entities related to the *fma:Right_precentral_gyrus* using the *fma:regional_part_of* or *fma:constitutional_part_of* object properties were extracted from the FMA using the following vSparQl request:

CONSTRUCT { ?x ?y ?z }

FROM <http://purl.org/sig/fma>

FROM NAMEDEV <rpo_precentral_gyrus> [

CONSTRUCT

{ temp:set temp:member ?x. }

FROM <http://purl.org/sig/fma>

WHERE {

fma:Right_precentral_gyrus gleen:OnPath(“([fma:regional_part_of]|[fma:constitutional_part_of])^*^” ?x). }]

WHERE GRAPH { <rpo_precentral_gyrus> { ?x ?y ?z }}.

This request was processed by a web service based on a local server at the university of Rennes 1. The result of this query (cf. Figure 2) lists all anatomical concepts from the right precentral gyrus to the human body entities illustrating part-whole relationships in FMA and was expressed using the Resource Description Framework^10^ (RDF).

**Figure 3:**
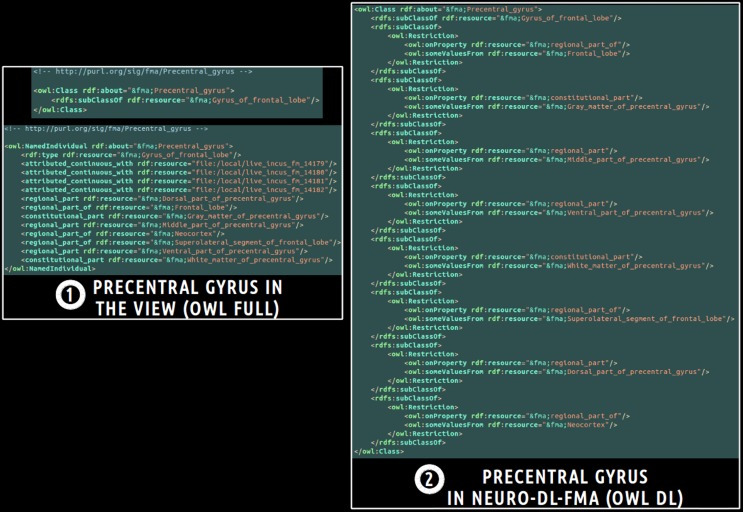
**Example of translation of an entity from a subset (or a “view”) of the Foundational Model of Anatomy (FMA) expressed in OWL Full (cf. part 1) into the corresponding NEURO-DL-FMA entity expressed using the OWL DL sublanguage (cf. part 2).** The “fma” prefix denotes that the entity was part of the FMA. In the left part of the figure (cf. part 1), concepts such as *fma:Precentral_gyrus* were both class and instance. Moreover, part-whole relationships such as *fma:constitutional_part* or *fma:regional_part_of* were expressed at the individuals' level. In the right part of the figure (cf. part 2), the same relationships were represented at the classes' level using existential restrictions.

Finally, a translation into OWL DL was necessary in order to enable the subsequent use of reasoning engines. This was achieved using a local java program based on the OWL API package^11^. All classes and object properties of the view were included in the ontology. As object properties were expressed at the individuals' level in the view, all object properties were translated at the classes' level using existential restrictions. Figure 3 depicts an example of translation of the *fma:Precentral_gyrus* entity from the view expressed in OWL Full (cf. part 1 of the figure) into NEURO-DL-FMA expressed in the OWL DL formalism (cf. part 2). In the view, entities such as *fma:Precentral_gyrus* appear both as a class and as an individual (cf. part 1). While part-whole relationships such as *fma:constitutional_part* or *fma:regional_part_of* were expressed at the individuals' level in the view (cf. part 1), the same object properties were expressed at the classes' level using existential restrictions in NEURO-DL-FMA (cf. part 2).

#### 2.1.3. The human connectomics ontology

The “Human Connectomics Ontology” (HCO) was created in order to represent brain regions, nearest neighbor topology and connectivity relationships assessed by diffusion tractography.

Different classes and object properties were defined in the HCO (cf. Tables 1, 2):

- *hco:Gray_matter_part*: Any cell part cluster constituting a part of (i.e., *fma:constitutional_part_of*, *fma:regional_part_of*) a gray matter region. This concept is more general than the *gray-matter-region*^12^ term defined in the FMC thesaurus (Swanson and Bota, 2010), because it is not grounded on criteria based on cytoarchitecture as the spatial distribution of a specific set of neuron types.
- *hco:MR_Node*: This concept denotes any *hco:Gray_matter_part* in brain images where a connection assessed by diffusion tractography begins or ends. This concept is an adaptation of the *node*^13^ term, defined in the FMC thesaurus (Swanson and Bota, 2010), dedicated to MRI datasets.
- *hco:White_matter_part*: Any cell part cluster constituting a part (i.e., *fma:constitutional_part_of*, *fma:regional_part_of*) of a white matter region.
- *hco:MR_Route*: Any physical route of white matter fiber bundles reconstructed by diffusion tractography that links two *hco:MR_Node* in brain images. This concept is an adaptation of the *route*^14^ term, defined in the FMC thesaurus (Swanson and Bota, 2010), dedicated to the brain images domain as diffusion tractography does not probe the path of white matter bundles directly, but water diffusion in brain.
- *hco:is_tracto_connected*: Object property that links an *hco:MR_Node* to an *hco:MR_Route*. The inverse property is *hco:tracto_connects*.
- *hco:mr_connection*: Symmetric object property that denotes the existence of a pathway assessed by diffusion tractography linking two different *hco:MR_Node*. If *a hco:is_tracto_connected b* and *b hco:tracto_connects c*, then *a hco:mr_connection c*. This property is an adaptation of the *connection*^15^ term, defined in the FMC thesaurus (Swanson and Bota, 2010), dedicated to connections assessed by diffusion tractography.
- *hco:continuous_with*: Symmetric object property that links an *fma:Anatomical_structure* to some nearest neighbor *fma:Anatomical_structure*.

**Table 1:** **Definition of Human Connectomics Ontology (HCO) classes**.

**Entity name**	**Property: subClassOf**	**Property: equivalent to**
hco:Gray_matter_part	fma:Region_of_cell_part_cluster_of_neuraxis	fma:Gray_matter_of_neuraxis and [(fma:constitutional_part_of some fma:Anatomical_structure) or (fma:regional_part_of some fma:Anatomical_structure)]
hco:White_matter_part	fma:Region_of_cell_part_cluster_of_neuraxis	fma:White_matter_of_neuraxis and [(fma:constitutional_part_of some fma:Anatomical_structure) or (fma:regional_part_of some fma:Anatomical_structure)]
hco:MR_Node	hco:Gray_matter_part	hco:Gray_matter_part and (hco:is_tracto_connected some hco:MR_Route)
hco:MR_Route	hco:White_matter_part	hco:White_matter_part and (hco:tracto_connects some hco:MR_Node)

The “fma:” prefix denotes terms originating from the Foundational Model of Anatomy. The “hco:” prefix denotes terms defined in the HCO.

**Table 2:** **Object properties of the human connectomics ontology**.

**Object property name**	**Domain**	**Range**	**Other properties**
hco:is_tracto_connected	hco:MR_Node	hco:MR_Route	Inverse: hco:tracto_connects
hco:mr_connection	hco:MR_Node	hco:MR_Node	Inverse: hco:mr_connection
hco:continuous_with	fma:Anatomical_structure	fma:Anatomical_structure	Inverse: hco:continuous_with
hco:part_of	fma:Anatomical_structure	fma:Anatomical_structure	Transitive. Super property of fma:regional_part_of and fma:constitutional_part_of
hco:part	fma:Anatomical_structure	fma:Anatomical_structure	Transitive. Super property of fma:regional_part and fma:constitutional_part

The “hco:” prefix denotes terms of the human connectomics ontology. The “fma:” prefix denotes terms originating from the Foundational Model of Anatomy.

**Figure 4:**
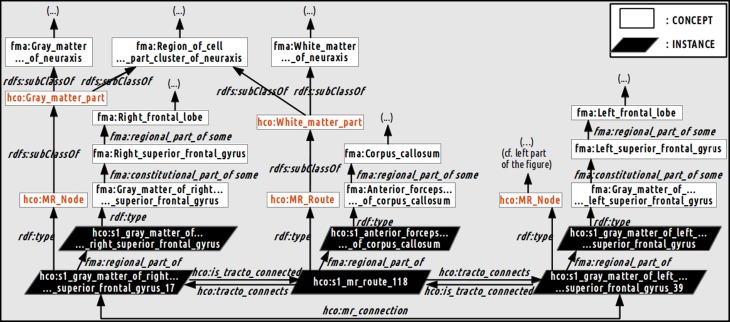
**Schematic illustration of how the relationships between the gray and white matter entities were represented in the Human Connectomics Ontology (HCO).** The “fma:” prefix denotes concepts (black) and object properties of the Foundational Model of Anatomy (FMA). The “hco:” prefix denotes concepts (red), instances and object properties of the HCO. The *hco:s1_gray_matter_of_right_superior_frontal_gyrus_17* instance of the *hco:MR_Node* class denotes a high resolution cortical parcel linked to the left hemisphere via the *hco:s1_mr_route_118* instance of the *hco:MR_Route* class. This instance was related to the anterior part of the corpus callosum via the *fma:regional_part_of* object property. The two different cortical parcels were linked via the *hco:mr_connection* object property. Part-whole relationships were represented thanks to the *fma:regional_part_of* and *fma:constitutional_part_of* object properties.

Figure 4 illustrates how a fiber bundle reconstructed by diffusion tractography that connected two cortical parcels via the corpus callosum white matter fiber bundle were represented using terms of the HCO. The *hco:s1_gray_matter_of_right_superior_frontal_gyrus_17* instance of the *hco:MR_Node* class denotes a high resolution cortical parcel. Part-whole relationships were represented thanks to the *fma:regional_part_of* and the *fma:constitutional_part_of* object properties: this latter cortical parcel was a regional part of some gray matter of the right superior frontal gyrus which was a constitutional part of the right superior frontal gyrus which was a regional part of the right frontal lobe. The parcel in the right hemisphere was linked to the other hemisphere via the *hco:s1_mr_route_118* instance of the *hco:MR_Route* class. This instance was related to the anterior part of the corpus callosum via a *fma:regional_part_of* object property. Finally the two different cortical parcels were linked via the *hco:mr_connection* object property.

### 2.2. Experimental work

**Figure 5:**
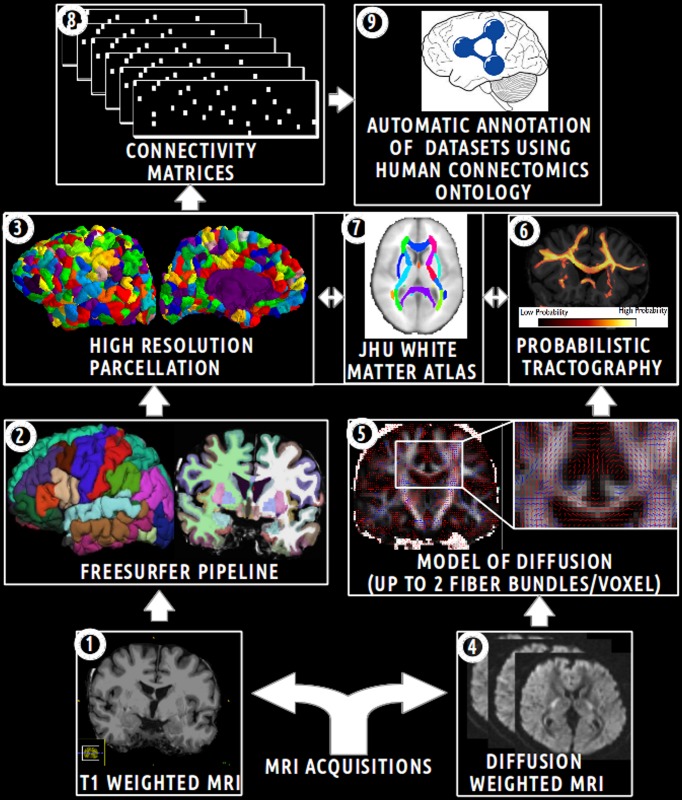
**Schematic overview of the main steps of the experimental work.** (1) Acquisition of T1 weighted MR images. (2) Automatic segmentation of brain regions using the Freesurfer pipeline. (3) Computation of an high resolution parcellation (CMTK toolkit). (4) Acquisition of diffusion weighted MR images. (5) Computation of diffusion model (FSL toolkit). (6) Computation of probabilistic tractography. (7) Automatic segmentation of anatomical fiber bundles based on the JHU white atlas (FSL toolkit). (8) Computation of connectivity matrices. (9) Automatic annotation of MRI connectomics datasets using terms of the Human Connectomics Ontology (HCO).

The aim of this experimental work was to assess how the HCO can effectively be used to enhance multi-level hypothesis-driven analysis of connectomics datasets. Figure 5 depicts an overview of the main steps of this experimental work.

#### 2.2.1. Neuroimaging data and preprocessing

The analysis was performed for five subjects of the NMR public database (Poupon et al., 2006). This database provided T1 (voxel size 0.9 × 0.9 × 1.2 mm) and diffusion-weighted datasets (voxel size of 1.9 × 1.9 × 2.0 mm) acquired with a GE Healthcare Signa 1.5 Tesla Excite II scanner. The diffusion datasets presented a high angular resolution (HARDI) based on 200 directions and a *b*-value of 3000 s/mm^2^. The use of a twice refocusing spin echo technique was used to compensate the echoplanar distortions due to eddy currents (Reese et al., 2003), at the first order. Susceptibility artifacts were corrected using a phase map acquisition. See “MRI acquisitions” on Figure 5.

The Freesurfer pipeline was applied on T1-weighted datasets producing cortical (Desikan et al., 2006) and sub-cortical segmentations (Dale et al., 1999). Then a *high resolution cortical parcellation* was performed for each subject. Each of the Freesurfer cortical regions was arbitrarily subdivided into a set of small and compact parcels of about 1.5 cm^2^ (Hagmann et al., 2008), resulting in 1000 parcels covering the entire cortex thanks to the connectome mapping toolkit^16^ (CMTK) (Daducci et al., 2012). The nearest neighbors of each parcel were assessed for each subject using a dilatation-based strategy. Thus, a total of 1000 cortical parcels and other regions (i.e., thalamus, caudate, putamen, pallidum, accumbens area, amygdala, hippocampus in both hemispheres, and brain-stem) were defined in the Freesurfer structural space. See “Freesurfer pipeline” and “high resolution parcellation” on Figure 5.

Two target masks were defined for the tractography in the Freesurfer structural space. The first target mask was defined as the set of high resolution cortical parcels included in the right medial Brodmann Area 6 (BA6). This right medial BA6 region of interest was defined in restricting the Freesurfer segmentation corresponding to the cortical region of the right superior frontal gyrus (i.e., “ctx-rh-superiorfrontal”) from *y* = −22 to *y* = 30 (MNI coordinates in the anteroposterior direction) and from the cingulate sulcus to the dorsal surface of the brain in order to include only voxels belonging to the gray matter on the medial wall (Johansen-Berg et al., 2004). The registration between the Freesurfer conformed space and the MNI space was computed using a linear registration (12 DOF) based on mutual information. This was achieved using the FLIRT tool of the FSL toolbox^17^. Finally, the second target mask was defined as the remaining cortical parcels or regions.

All the 20 white matter tracts of the JHU white matter tractography probabilistic atlas based on diffusion tensor imaging (Wakana et al., 2007; Hua et al., 2008) were segmented using a threshold at 25: anterior radiation of thalamus, corticospinal tract, anterior segment of cingulum bundle, anterior forceps of corpus callosum, posterior forceps of corpus callosum, inferior occipitofrontal fasciculus, inferior longitudinal fasciculus, uncinate fasciculus, superior longitudinal fasciculus in both hemispheres. A total of 22 white matter masks (one for each fiber bundle and two for the rest of the white matter in both hemispheres) were defined as seed masks for the tractography. An automatic *linear* (12 DOF) registration based on the correlation ratio between the JHU white matter tractography atlas and the Freesurfer structural space was computed using the FLIRT tool. See “JHU white matter atlas” on Figure 5.

A registration between the Freesurfer structural space and the diffusion dataset space was computed using a *rigid registration* (6 DOF) based on mutual information implemented in FLIRT. The registration was performed considering the average of five *B0 volumes* (i.e., volumes with *b*-value = 0) and the brain volume in the Freesurfer structural space.

#### 2.2.2. Connectivity assessed by diffusion tractography

The aim of the *probabilistic tractography* was to characterize the connectivity pattern of each structural element, denoted by seeds, in probing the Brownian movement of water molecules within white matter fiber bundles. Probabilistic tractography was performed using the *bedpostX* and *probtrackX2* tools, part of the FSL toolbox (Behrens et al., 2007). *BedpostX* uses a Monte Carlo Markov chain sampling to estimate the diffusion parameters at each voxel. The probabilistic tractography could model up to two fiber bundles in each voxel. The burn-in of the Markov chains was set to 3000 in order to ensure convergence of the model. See “model of diffusion” on Figure 5.

A whole brain *probabilistic tractography* was achieved in order to assess gray-to-gray connectivity between the two target masks defined above. The *probtrackX2* tractography tool drew 5000 probabilistic streamlines that were sent in both directions from the distribution connectivity of each white matter seed voxel. The 22 white matter masks defined above were used successively as seed for the tractography. If the streamline hitted the two target masks at two locations along either sides of the streamline, then the corresponding row and column of the connectivity matrix was filled. Streamlines that stopped before reaching a length of 30 mm or that passed through an exclusion mask (i.e., ventricles, cortico-spinal fluid (CSF), the choroid-plexus) were discarded. A distance correction was used in order to correct the fact that connectivity distribution drops with distance from the seed voxel. See “probabilistic tractography” on Figure 5.

Each of the 22 voxel-wise connectivity matrices was converted into a region-wise connectivity matrix (11 × 1004) between the 11 cortical regions in the first target mask and the 1004 cortical and other brain regions in the second target mask. The connectivity between two regions in the region-wise connectivity matrix was computed as the mean of the connectivities between the voxels belonging to the corresponding regions. After a logarithmic transformation of the region-wise connectivity matrix, a normalization of the values of each row was achieved. Finally, connectivity values in the region-wise connectivity matrices were thresholded at 0.7 in order to keep only connections reconstructed by diffusion tractography with a high probability. See “connectivity matrices” on Figure 5.

#### 2.2.3. Automatic annotation of MRI connectomics datasets

The HCO was populated with instances describing fiber bundles assessed by diffusion tractography between different gray matter regions of the five healthy subjects. This was achieved using a Java program based on OWL API. First, each gray matter region was represented as an instance of the *hco:Gray_matter_part* class. If the gray matter region was a high resolution cortical parcel, then the cortical parcel was related to the instance of the overlapping gyrus via the *fma:regional_part_of* object property. If two instances of *fma:Anatomical_structure* were found to be nearest neighbors then they were related together using the *hco:continuous_with* object property. Finally, each binary region-wise connectivity matrix (11 × 1004) was used to encode the connectivity reconstructed by diffusion tractography between the 11 cortical regions defined in the first target mask and the 1004 brain regions defined in the second target mask (cf. Figure 5, “automatic annotation of datasets using HCO”):

- The two corresponding structural elements of the row and column were represented as instances of the *hco:MR_Node* class and were related together using the *hco:mr_connection* object property.
- An instance of the *hco:MR_Route* class was created and related to the two corresponding instances of the *hco:MR_Node* class using the *hco:tracto_connects* object property.
- If the connectivity matrix was associated with an anatomical white matter fiber bundle, then the instance of the *hco:MR_Route* class was related to the instance of the overlapping fiber bundle using the *fma:regional_part_of* object property.

#### 2.2.4. Answering competency questions through the human connectomics ontology

Table 3 presents each competency question translated into terms of the HCO before submission to the FaCT++ reasoning engine via the “DL Query” tab of the ontology editor Protégé (Rubin et al., 2007). FaCT++ is an efficient tableaux-based reasoner implemented using C++ that supports OWL DL. It is used as one of the default reasoners in Protégé (version 4). In order to find all parts of (*fma:regional_part_of*, *fma:constitutional_part_of*) an anatomical structure, the *hco:part_of* transitive object property was used. The right medial parietal cortex was expressed using a conjunction of terms from the ontology: *fma:Cortex_of_right_parietal_lobe* and *fma:Medial_segment_of_cerebral_hemisphere*. The inferior frontal cortex was translated into the *fma:Orbitobasal_segment_of_right_frontal_lobe* term.

**Table 3:** **Translation of the four competency questions into Description Logic (DL) queries using terms of the Human Connectomics Ontology (HCO)**.

**Competency Questions (CQ)**	**DL queries using terms of the HCO**
CQ1: which gray matter parts of the right superior frontal gyrus have a connectivity pattern passing through the corticospinal tract or through some gray matter parts of the right precentral gyrus?	Query1: (part_of some Right_superior_frontal_gyrus) and ((is_tracto_connected some (part_of some Right_corticospinal_tract_of_brain)) or (mr_connection some (part_of some Right_precentral_gyrus)))
CQ2: which gray matter parts of the right superior frontal gyrus have a connectivity pattern passing through some gray matter parts of the right medial parietal cortex or through some gray matter parts of the inferior frontal cortex?	Query2: (part_of some Right_superior_frontal_gyrus) and (mr_connection some ((part_of some Cortex_of_right_parietal_lobe) and (part_of some Medial_segment_of_cerebral_hemisphere)) or mr_connection some (part_of some Orbitobasal_segment_of_right_frontal_lobe))
CQ3: which gray matter parts of the right superior frontal gyrus have a connectivity pattern passing through the corticospinal tract or through some gray matter parts of the right precentral gyrus or through some gray matter parts contiguous with the right precentral gyrus?	Query3: (part_of some Right_superior_frontal_gyrus) and ((is_tracto_connected some (part_of some Right_corticospinal_tract_of_brain)) or (mr_connection some (part_of some Right_precentral_gyrus)) or (mr_connection some (continuous_with some (part_of some Right_precentral_gyrus))))
CQ4: which anatomical white matter fiber bundles connect some gray matter parts of the right superior frontal gyrus to some gray matter parts of the right temporal lobe?	Query4: part some ((tracto_connects some (part_of some Right_temporal_lobe)) and (tracto_connects some (part_of some Right_superior_frontal_gyrus)))

## 3. Results

### 3.1. Right medial brodmann area 6 region of interest

Our definition of the right medial BA6 was decomposed into eleven high resolution cortical parcels numbered: 20, 12, 32, 9, 17, 13, 42, 18, 24, 25, 38 in all five subjects. Columns 1 and 2 of the **Figure 8** depict a map of these parcels on a medial view of the gray/white interface of the right hemisphere.

### 3.2. Structural connectivity assessed by diffusion tractography

Table 4 summarizes the set of regions that were found connected to the right medial BA6 via fiber bundles assessed by diffusion tractography for each subject. This set of regions was expressed using FMA terms denoting regions at a high level of granularity (i.e., gyrus level, or other anatomical structures): as an illustration the *hco:s1_gray_matter_of_left_superior_frontal_gyrus_39* entity, which was a cortical region of the left superior frontal gyrus (cf. Figure 4), was denoted using the *fma:Left_superior_frontal_gyrus* concept. 23.7% of the total of anatomical terms that were connected to the right medial BA6 in our data were found common to the five subjects. 38.9% (69.5% resp.) of these anatomical terms were found common to at least 4 (3 resp.) subjects.

**Table 4:** **Connectivity assessed by diffusion tractography between the right medial Brodmann area 6 (BA6) and other regions of the brain using the FMA terminology for the 5 subjects**.

**Subject ID**	**Gyri of the left hemisphere**	**Gyri of the right hemisphere**	**Other brain regions**
Subject01	Anterior part of left middle frontal gyrus, Caudal part of left anterior cingulate gyrus, Cortex of left insula, Left frontal pole, Left lateral orbital gyrus, Left medial orbital gyrus, Left posterior cingulate gyrus, Left precentral gyrus, Left superior frontal gyrus, Opercular part of left inferior frontal gyrus, Triangular part of left inferior frontal gyrus	Anterior part of right middle frontal gyrus, Caudal part of right anterior cingulate gyrus, Cortex of right insula, Isthmus of right cingulate gyrus, Opercular part of right inferior frontal gyrus, Orbital part of right inferior frontal gyrus, Right frontal pole, Right inferior temporal gyrus, Right lateral occipital gyrus, Right lingual gyrus, Right medial orbital gyrus, Right middle temporal gyrus, Right posterior cingulate gyrus, Right precuneus, Right superior frontal gyrus, Right superior parietal lobule, Rostral part of right anterior cingulate gyrus, Triangular part of right inferior frontal gyrus	Brainstem, Left globus pallidus, Left putamen, Left thalamus, Right globus pallidus, Right putamen, Right thalamus
Subject02	Anterior part of left middle frontal gyrus, Caudal part of left anterior cingulate gyrus, Left medial orbital gyrus, Left middle temporal gyrus, Left paracentral lobule, Left posterior cingulate gyrus, Left precentral gyrus, Left precuneus, Left superior frontal gyrus, Opercular part of left inferior frontal gyrus, Posterior part of left middle frontal gyrus, Rostral part of left anterior cingulate gyrus	Anterior part of right middle frontal gyrus, Caudal part of right anterior cingulate gyrus, Cortex of right insula, Isthmus of right cingulate gyrus, Opercular part of right inferior frontal gyrus, Right frontal pole, Right inferior temporal gyrus, Right middle temporal gyrus, Right paracentral lobule, Right posterior cingulate gyrus, Right precuneus, Right superior frontal gyrus	Brainstem, Left globus pallidus, Left putamen, Left thalamus, Right globus pallidus, Right putamen, Right thalamus
Subject03	Anterior part of left middle frontal gyrus, Left frontal pole, Left inferior parietal lobule, Left inferior temporal gyrus, Left medial orbital gyrus, Left middle temporal gyrus, Left posterior cingulate gyrus, Left superior frontal gyrus, Left supramarginal gyrus, Posterior part of left middle frontal gyrus	Anterior part of right middle frontal gyrus, Caudal part of right anterior cingulate gyrus, Isthmus of right cingulate gyrus, Opercular part of right inferior frontal gyrus, Orbital part of right inferior frontal gyrus, Right inferior parietal lobule, Right inferior temporal gyrus, Right lateral occipital gyrus, Right middle temporal gyrus, Right paracentral lobule, Right posterior cingulate gyrus, Right precentral gyrus, Right precuneus, Right superior frontal gyrus, Right supramarginal gyrus, Rostral part of right anterior cingulate gyrus, Triangular part of right inferior frontal gyrus	Brainstem, Left putamen, Left thalamus, Right caudate nucleus, Right globus pallidus, Right putamen, Right thalamus
Subject04	Anterior part of left middle frontal gyrus, Caudal part of left anterior cingulate gyrus, Cortex of left insula, Left frontal pole, Left inferior parietal lobule, Left inferior temporal gyrus, Left lateral occipital gyrus, Left medial orbital gyrus, Left postcentral gyrus, Left posterior cingulate gyrus, Left precentral gyrus, Left superior frontal gyrus, Left superior parietal lobule, Left superior temporal gyrus, Left supramarginal gyrus, Opercular part of left inferior frontal gyrus, Orbital part of left inferior frontal gyrus, Triangular part of left inferior frontal gyrus	Anterior part of right middle frontal gyrus, Caudal part of right anterior cingulate gyrus, Cortex of right insula, Orbital part of right inferior frontal gyrus, Right frontal pole, Right inferior parietal lobule, Right lateral occipital gyrus, Right lingual gyrus, Right medial orbital gyrus, Right paracentral lobule, Right postcentral, Right posterior cingulate gyrus, Right precentral gyrus, Right precuneus, Right superior frontal gyrus, Right supramarginal gyrus, Triangular part of right inferior frontal gyrus	Brainstem, Left globus pallidus, Left putamen, Left thalamus, Right caudate nucleus, Right globus pallidus, Right putamen, Right thalamus
Subject05	Anterior part of left middle frontal gyrus, Caudal part of left anterior cingulate gyrus, Cortex of left insula, Left frontal pole, Left fusiform gyrus, Left inferior temporal gyrus, Left lateral orbital gyrus, Left medial orbital gyrus, Left paracentral lobule, Left posterior cingulate, Left precentral gyrus, Left superior frontal gyrus, Posterior part of left middle frontal gyrus, Triangular part of left inferior frontal gyrus	Anterior part of right middle frontal gyrus, Caudal part of right anterior cingulate gyrus, Right frontal pole, Right inferior parietal lobule, Right inferior temporal gyrus, Right lateral occipital gyrus, Right lateral orbital gyrus, Right lingual gyrus, Right medial orbital gyrus, Right middle temporal gyrus, Right paracentral lobule, Right posterior cingulate gyrus, Right precentral gyrus, Right precuneus, Right superior frontal gyrus, Right supramarginal gyrus, Rostral part of right anterior cingulate gyrus	Brainstem, Left caudate nucleus, Left putamen, Left thalamus, Right caudate nucleus, Right globus pallidus, Right putamen, Right thalamus

### 3.3. Semantic annotation of MRI connectomics datasets

The HCO and its NEURO-DL-FMA module contained 811 classes, 11 object properties, no data property and a mean of 2321 instances per subject. The HCO was classified in less than 6 s per subject using the FaCT++ reasoning engine on a dual core processor, 3.06 GHz, 3.9 Go RAM workstation. The reasoning engine was used both to keep ontologies in a logically consistent state, and to infer new axioms between brain regions.

**Figure 6:**
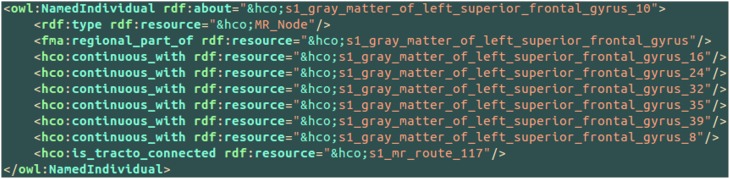
**Example of use of some part-whole (i.e., *fma:regional_part_of*), spatial (i.e., *hco:continuous_with*) and connectivity (i.e., *hco:is_tracto_connected*) relationships between different cortical parcels (i.e., *hco:MR_Node*) and a fiber bundle reconstructed by diffusion tractography (i.e., *s1_mr_route_117*) with terms of the Human Connectomics Ontology (HCO).** The “fma” and “hco” prefixes denote entities of the Foundational Model of Anatomy (FMA) and of the HCO, respectively.

An example of the use of some part-whole, spatial and connectivity relationships using the HCO terms is provided on Figure 6. The part-whole relationship was expressed using the *fma:regional_part_of* object property denoting the fact that the high resolution cortical parcel 10 (i.e., *hco:s1_gray_matter_of_left_superior_frontal_gyrus_10*) was a regional part of the gray matter of the left superior frontal gyrus of the subject 01. The spatial relationship was expressed thanks to the *hco:continuous_with* object property denoting the fact that the cortical parcel 10 had several nearest neighbors in the gray matter of the left frontal gyrus, namely parcels number 16, 24, 32, 35, 39, and 8. Finally, the cortical parcel 10 was linked to the instance number 117 of the *MR_Route* class with the *hco:is_tracto_connected* object property denoting the existence of a fiber bundle reconstructed by diffusion tractography.

**Figure 7:**
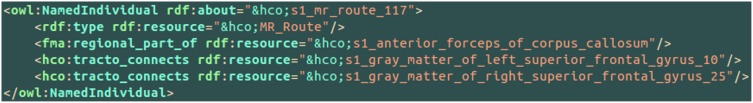
**Illustration of the use of the *hco:tracto_connects* connectivity relationship between a fiber bundle assessed by diffusion tractography (i.e., *hco:MR_Route*) and different cortical parcels (i.e., *hco:MR_Node*) using terms of the Human Connectomics Ontology (HCO).** The “fma” and “hco” prefixes denote entities of the Foundational Model of Anatomy (FMA) and of the HCO, respectively. The object property *fma:regional_part_of* denotes a part-whole relationship.

Figure 7 aimed at illustrating the use of the *hco:tracto_connects* connectivity relationship using HCO terms. The *s1_mr_route_117* instance of the *MR_Route* class denoted a fiber bundle assessed by diffusion tractography belonging to the subject 01. This route 117 was linked to two cortical parcels in the left (number 10) and right (number 25) superior frontal gyri using the *hco:tracto_connects* object property. The *fma:regional_part_of* object property expressed the fact that the route 117 was a part of the anterior forceps of the corpus callosum.

### 3.4. Ontology application testing: the medial BA6 case study

Table 5 summarizes the results of the queries expressing our competency questions (cf. 2.1.1) and using terms of the HCO before submission to the FaCT++ reasoning engine. These results are expressed using the high resolution cortical parcel identifiers for columns 2, 3, and 4 (i.e., 17 was the identifier of the cortical parcel denoted by the following instance: *hco:s1_gray_matter_of_right_superior_frontal_17*). Column 5 of the table lists a set of instances denoting the white matter fiber bundles that were found to match the criteria of the last competency question.

**Table 5:** **Results of the different queries corresponding to the Competency Questions (CQ) that were translated into terms of the Human Connectomics Ontology (HCO) and submitted to the FaCT++ reasoning engine (cf. Table 3)**.

**Subject ID**	**Cortical parcels matching the query 1 criteria**	**Cortical parcels matching the query 2 criteria**	**Cortical parcels matching the query 3 criteria**	**Cortical parcels matching the query 4 criteria**
Subject01	17	18, 20, 24, 25, 38, 42	12, 17, 32	s1_right_superior_longitudinal_fasciculus
Subject02		24, 25, 42	12, 17, 20	s2_right_superior_longitudinal_fasciculus
Subject03	9, 17	13, 18, 20, 24, 25, 38, 42	9, 12, 17, 18, 32	s3_right_superior_longitudinal_fasciculus
Subject04	9, 12, 17, 20, 24, 32, 42	38, 42	9, 12, 17, 20, 24, 32, 42	
Subject05	9, 12, 13, 17, 20, 32, 42	9, 12, 13, 18, 20, 24, 25, 32, 38, 42	9, 12, 13, 17, 20, 32, 42	s5_right_superior_longitudinal_fasciculus, s5_right_inferior_longitudinal_fasciculus

CQ1: Which gray matter parts of the right superior frontal gyrus have a connectivity pattern passing through the corticospinal tract or through some gray matter parts of the right precentral gyrus?

CQ2: Which gray matter parts of the right superior frontal gyrus have a connectivity pattern passing through some gray matter parts of the right medial parietal cortex or through some gray matter parts of the right inferior frontal cortex of the frontal lobe?

CQ3: Which gray matter parts of the right superior frontal gyrus have a connectivity pattern passing through the corticospinal tract or through some gray matter parts of the right precentral gyrus or through some gray matter parts contiguous with the right precentral gyrus?

**Figure 8:**
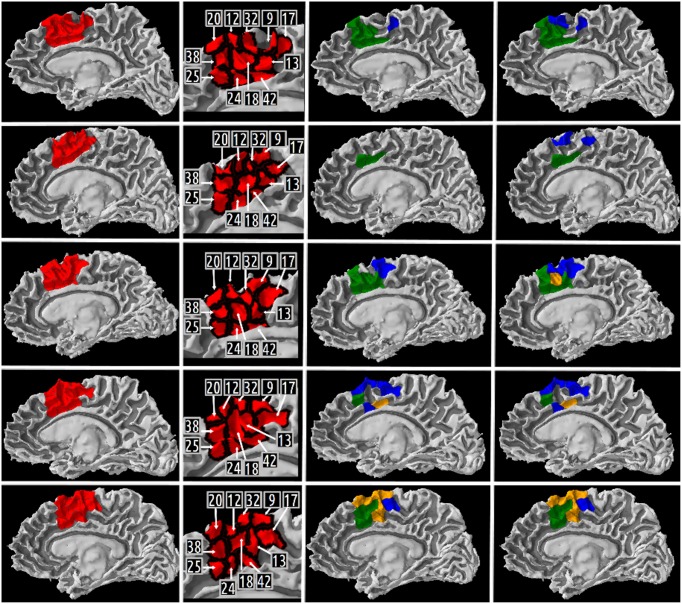
**Medial view of the right gray/white interface of the five subjects.** Column 1 represents in red the medial Brodmann area 6 region of interest for subject 01, 02, 03, 04, 05, respectively. Column 2 depicts a map of the different high resolution cortical parcels that were parts of the region of interest and the corresponding identifiers. The two last columns represent the results of three different queries corresponding to some of our Competency Questions (CQ) that were translated into terms of the Human Connectomics Ontology (HCO) and submitted to the FaCT++ reasoning engine (cf. Table 3). Column 3 represents in blue (resp. green) the cortical parcels matching the query 1 (resp. 2) criteria (cf. Table 5). When a parcel was the result of both queries, it was represented in orange. Column 4 represents in blue the cortical parcels matching the query 3 criteria (cf. Table 5). On column 3, the same color code was kept for the green and orange cortical parcels.

CQ4: Which anatomical white matter fiber bundles connect some gray matter parts of the right superior frontal gyrus to some gray matter parts of the right temporal lobe? See the Figure 8 for a graphic representation of these results.

A map of the different high resolution cortical parcels presented in Table 5 in columns 2, 3, and 4 were plotted on the gray/white interface of the right hemisphere for each subject (cf. Figure 8). The first column of this figure represents (in red) the right medial BA6 region of interest for each subject. The second column of the figure depicts a map of the different cortical parcels that are parts of the region of interest and the corresponding identifiers. The third column represents (in blue and green) the results summarized in columns 2 (CQ1 query) and 3 (CQ2 query) of Table 5, respectively. The fourth column of the figure represents (in blue and green) the results summarized in columns 3 and 4 of Table 5, respectively. The cortical parcels that were found to meet the criteria of several competency questions were represented in orange color.

## 4. Discussion

The aim of this study was to design an ontology for *in vivo* human connectomics, i.e., suitable to describe connectomics data revealed by MRI, thus facilitating their retrieval, sharing and comparison with other neuroscience knowledge resources. A new ontology was created, called the “Human Connectomics Ontology” (HCO), that models brain regions and connectivity relationships assessed by diffusion tractography, using a three step methodology. First, the domain of discourse was specified using a set of competency questions grounded on the paradigmatic medial BA6 case study. Then, the HCO was based on a neuroanatomical ontology module called “NEURO-DL-FMA” in order to represent gross anatomy of the brain (i.e., gray matter regions, white matter fiber bundles). Finally, a set of entities was explicitly defined in the HCO to represent some aspects of the connectivity that could be observed through diffusion MRI. Moreover, an experimental work was achieved in order to show how the HCO could be effectively used to express complex queries and process them using a DL reasoning engine.

### 4.1. A neuroanatomical ontology for *in vivo* human connectomics

The medial BA6 case study provided an interesting use case for expressing competency questions for the specification of the HCO. In comparing macaque to human brain, Johansen-Berg et al. showed how the medial BA6 could be subdivided into two major anatomo-functional regions—Supplementary Motor Area (SMA) and pre-SMA—using distinct long-range connectivity patterns assessed by diffusion tractography (Johansen-Berg et al., 2004). As these connectivity patterns were concerned with rich neuroanatomical concepts denoting regions at different levels of resolution (e.g., “part of superior frontal gyrus,” “part of medial parietal cortex,” etc.), our set of competency questions (Neuhaus and Vizedom, 2013), inspired by this study, involved multi-level analysis and rich neuroanatomical expressivity.

NEURO-DL-FMA, defined as a neuroanatomical ontology of the gross-anatomy of the brain, was first extracted as a view from the FMA reference ontology in OWL Full and then translated into OWL DL. Different studies tried to convert the entire FMA (Protege frames version) into different OWL versions and to use reasoning engines (Golbreich et al., 2006; Golbreich et al., 2013). Our strategy was to extract from the OWL Full version of FMA a “view” of the brain constituted only of entities which were parts of the neuraxis, following (Turner et al., 2010; Shaw et al., 2011). Though the latter study achieved brain image analysis using the *DXBrain* software (Detwiler et al., 2009), no DL reasoning engine was used, however.

The HCO was designed in taking into account both the reference ontology in neuroanatomy FMA and the conceptual framework of structural connectivity FMC. If NEURO-DL-FMA was clearly grounded on a subset of FMA, however no concept of the BAMS^18^ ontology was used in the HCO. Indeed, structural connectivity addressed in BAMS primarily concerns pathway-tracing experiments in animals, whereas we were focusing on connectivity as observed in diffusion MRI. Nevertheless, the FMC was a useful source of inspiration. Some terms (i.e., *gray-matter-region*^19^, *node*^20^, *route*^21^, *connection*^22^) of the FMC thesaurus were instrumental in the definition of some new HCO entities (i.e., *hco:Gray_matter_part*, *hco:MR_Node*, *hco:MR_Route*, *hco:mr_connection*) dedicated to MRI connectomics.

### 4.2. Experimental work

The experimental work was achieved in order to illustrate how the semantic annotation and the reasoning about MRI connectomics datasets could enhance the analysis of connectivity patterns present in this data. Connectivity was assessed using a probabilistic tractography method in the living human brain. It is worth saying that tractography results should be interpreted with care (Jones et al., 2013). Indeed, anatomical connectivity denotes the white matter fibers which physically connect brain regions, whereas connectivity assessed by diffusion tractography relies on water diffusion as an indirect probe of axon geometry. In fact, tractography infers fiber bundles pathways through the diffusion field in assuming that the direction of least hindered diffusion is aligned with axons (Jbabdi and Johansen-Berg, 2011). If this hypothesis seems reasonable at the axon level (microscopic scale), it has several practical consequences at the imaging level (macroscopic scale). For example, complex microscopic architectures of white matter fibers are often oversimplified by local models of axons-diffusion mapping. Moreover, tractography algorithms cannot determine with accuracy the origin and the termination of connections in the cortex (Jbabdi and Johansen-Berg, 2011). Thus, these different ambiguities combined with imaging noise generate spurious connections between brain regions. This is why it is so important to describe such connections using conceptual entities that allow distinguishing them from connections observed using tracer-based methods, e.g., collated in the BAMS database. Furthermore, although probabilistic tractography methods do not estimate the connection strength between two regions (as tracer-based methods actually do), they allow assessing the confidence in the pathway of least hindrance to diffusion. This is a major advantage of probabilistic tractography over deterministic tractography, since the latter cannot provide such confidence cues. So, although tractography is limited by several biases, it is currently the only available tool that gives us the opportunity to investigate anatomical connectivity *non invasively* and in the *living* human brain.

Our automatic annotation of brain images was based on brain segmentations achieved thanks to the use of atlases such as the Freesurfer (Fischl et al., 2004; Desikan et al., 2006) or JHU white matter tractography atlas (Wakana et al., 2007; Hua et al., 2008). Such atlases should be used with care in case of brain pathology, however. Another approach for automatic probabilistic reconstruction of *in vivo* white matter bundles based on global tractography called “Tracula” seems more robust in presence of pathology (Yendiki et al., 2011). However, the JHU white matter tractography atlas was preferred in our experimental work, because FMA provided a one-to-one mapping with the terminology of this atlas. As this atlas provided a probability that a particular voxel belonged to a white matter bundle, some white matter voxels could be mislabelled particularly in case of two close white matter bundles. As an illustration, Table 5 column 4 gives the names of the anatomical white matter fiber bundles reconstructed by diffusion tractography which connected some gray matter parts of the right superior frontal gyrus to some gray matter parts of the right temporal lobe. Two different anatomical bundles were found in our data: the right superior longitudinal fasciculus (found in subjects 01, 02, 03, and 05) and the right inferior longitudinal fasciculus (found in subject 05). If the right superior longitudinal fasciculus was found anatomically relevant in the MRI atlas of human white matter (Mori et al., 2005), the right inferior longitudinal fasciculus was not, however. This spurious annotation may have resulted from some mislabelled white matter voxels, since superior and inferior longitudinal fasciculi appeared close to one another particularly in the occipital lobe of the brain.

The HCO ontology aimed at representing brain regions and connectivity relationships assessed by diffusion tractography in the living brain. This was achieved by creating the corresponding instances of the ontology classes in the annotation file. If the seed of the tractography was located in a segmented white matter bundle, then the instance representing the pathway generated by the tractography algorithm was related to the instance representing this anatomical white matter bundle using the *fma:regional_part_of* object property (cf. Figure 4). This is based on the assumption that the whole pathway of the tractography was located within the segmented white matter bundle, which would need to be verified.

### 4.3. Automatic inferences on brain connectivity

Automatic annotation of brain images with terms of an ontology and subsequent analysis using reasoning engines enable powerful information retrieval thanks to the high level representation of the image content embedded in the ontology. As an illustration, when an investigator queries through the HCO all cortical parts of the orbitobasal segment of the right frontal lobe which are connected to some medial BA6 parts via fiber bundles assessed by diffusion tractography, the reasoning engine takes advantage of both class-level knowledge (what are the gyri included in the orbitobasal segment of the right frontal lobe cortex?) and instance-level facts derived from image evidence (which data instantiate some regional parts of these gyri classes?).

Different initiatives used automatic inferences based on structured knowledge in order to represent cerebral connectivity: (1) Neurolex and (2) KEfED (Knowledge Engineering from Experimental Design) approach. (1) Neurolex is a semantic wiki-based website and knowledge management system dedicated to neurobiology whose primary goals are to assist neuroscientists in reviewing anatomical features, linking them to other neuroscience resources, and stimulating discussion with other scientists especially about controversial or missing features (Larson and Martone, 2013). Due to the fact that the semantic MediaWiki platform (on which Neurolex was built) did not support many of the first-order logic features that are needed to achieve OWL DL reasoning, the RDF version of Neurolex was deployed into an instance of the OWL-IM semantic repository (http://www.ontotext.com/owlim) providing SPARQL 1.1 querying capabilities. In Larson and Martone (2013), the authors demonstrated how a SPARQL query could retrieve from Neurolex “all brain regions that send projections into the cerebellum or any of its parts via mossy fibers.” In order to search recursively all subclasses which were regional parts of the cerebellum, the authors used the “*property paths*”^23^ feature of SPARQL 1.1. (2) Another initiative was based on a KEfED approach. First an experimental design was modeled as a workflow using a set of KEfED models which aimed at representing the experiment using structured information. Secondly, interpretations of the experimental observations were achieved using a domain-specific reasoning. In Russ et al. (2011), the authors illustrated the relevance of a KEfED approach through a neural connectivity use case based on tract-tracing experiments in animal subjects. Tract-tracing experiments consist of injecting in a site a chemical tracer which is then transported along neurons' axonal fibers. Interpretation of such tract-tracing experiments aims at describing connections between different brain regions. Spatial reasoning was used especially to process the part-whole and the overlaps relationships between regions. Basic geometric features were imported from the BAMS neuroanatomical ontology of the rat into PowerLoom, a first-order logic knowledge representation and closed-world reasoning system. Thus, the authors demonstrated how connectivity matrices could be inferred through the use of spatial reasoning and the modeling of the tract-tracing experiments using a KEfED approach. HCO and its NEURO-DL-FMA module differ from these approaches because they were expressed in the W3C standard OWL language and used the OWL DL description logics sublanguage. Moreover, the FaCT++ reasoning engine was used both to ensure the satisfiability of the ontologies, and to infer new axioms using transitive part-whole, spatial relationships, and connectivity relationships assessed by diffusion tractography. As a result, complex queries (cf. Table 3) could be formulated directly via the “DL Query” tab of the Protégé ontology editor, in a more expressive way than using the SPARQL language.

An interesting initiative dedicated to diffusion tractography called the “White Matter Query Language” (Wassermann et al., 2013) used a textual language in order to express anatomical descriptions of white matter tracts. In selecting streamlines from a whole brain deterministic tractography using both anatomical structure terms describing where streamlines end or pass through, relative position terms of streamlines from other anatomical structures and finally logical operations terms, Wassermann et al. used different expressions to define some association, projection and commissural tracts (Wassermann et al., 2013). Although the latter approach explicitly defined some white matter tracts using a near-to-English syntax, it did not provide an ontology in order to annotate results of *in vivo* human connectomics based on diffusion tractography nor reasoning capabilities in order to infer part-whole, spatial or connectivity relationships at different level of granularity.

## 5. Conclusion and perspectives

In this article we have described a neuroanatomical ontology dedicated to human connectomics called the Human Connectomics Ontology (HCO) that could represent brain regions and connectivity assessed by diffusion tractography in the living human brain. Moreover, an experimental work was achieved in order to show how the HCO could be effectively used within an information system to express complex queries concerning MRI connectomics datasets and process them using a DL reasoning engine. This approach can facilitate comparison of data across scales, modalities and species.

Future work will consist in the development of a visualization module in order to display macro-connectome at different levels of granularity in a matrix or a network form. This module could leverage the reasoning engine to retrieve connections assessed by diffusion tractography between different gray matter regions. To finish, a long-term goal will consist in facilitating the consistent querying of *in vivo* MRI connectomics data and tracer-based observations made in multiple species. This would be of major interest for assessing the validity of putative connections highlighted in human MR connectomics.

## Author contributions

TM and BG designed the work, analyzed data, drafted the work, approved the final version to be published and agreed to be accountable for all aspects of the work ensuring questions related to the accuracy or integrity of any part of the work.

### Conflict of interest statement

The authors declare that the research was conducted in the absence of any commercial or financial relationships that could be construed as a potential conflict of interest.

## Footnotes

^1^OWL, http://www.w3.org/TR/owl-ref/

^2^OWL DL, http://www.w3.org/TR/owl-ref/#OWLDL

^3^FaCT++, http://owl.man.ac.uk/factplusplus/

^4^Freesurfer, http://surfer.nmr.mgh.harvard.edu/fswiki

^5^FSL, http://fsl.fmrib.ox.ac.uk/fsl/fslwiki/FSL

^6^HCO, https://medicis.univ-rennes1.fr/activities/theme3/projects/semantic-annotation/index

^7^University of Washington's Structural Informatics Group, http://sig.biostr.washington.edu/

^8^MySQL, https://www.mysql.com/

^9^Jena, https://jena.apache.org/

^10^RDF, http://www.w3.org/TR/2014/REC-rdf11-concepts-20140225/

^11^OWL API, http://owlapi.sourceforge.net/

^12^Gray-matter-region: http://brancusi1.usc.edu/thesaurus/definition/gray-matter-region/

^13^Node: http://brancusi1.usc.edu/thesaurus/definition/node/

^14^Route: http://brancusi1.usc.edu/thesaurus/definition/route/

^15^Connection: http://brancusi1.usc.edu/thesaurus/definition/connection/

^16^CMTK, http://www.cmtk.org/

^17^FSL, http://fsl.fmrib.ox.ac.uk/fsl/fslwiki/FSL

^18^BAMS, http://brancusi1.usc.edu/ontology/

^19^Gray-matter-region, http://brancusi1.usc.edu/thesaurus/definition/gray-matter-region/

^20^Node, http://brancusi1.usc.edu/thesaurus/definition/node/

^21^Route, http://brancusi1.usc.edu/thesaurus/definition/route/

^22^Connection, http://brancusi1.usc.edu/thesaurus/definition/connection/

^23^Property paths, http://www.w3.org/TR/sparql11-query/#propertypaths

## References

[B1] Anwander A. Tittgemeyer M. von Cramon D. Y. Friederici A. D. Knösche T. R. (2007). Connectivity-based parcellation of broca's area. Cereb. Cortex 17, 816–825. 10.1093/cercor/bhk034 16707738

[B2] Baader F. Calvanese D. McGuinness D. Nardi D. Patel-Schneider P. (2003). The Description Logic Handbook: Theory, Implementation, and Applications. New York, NY: Cambridge University.

[B3] Basser P. J. Pajevic S. Pierpaoli C. Duda J. Aldroubi A. (2000). *In vivo* fiber tractography using dt-mri data. Magn. Reson. Med. 44, 625–632. 10.1002/1522-2594(200010)44:4<625::AID-MRM17>3.0.CO;2-O 11025519

[B4] Behrens T. E. J. Berg H. J. Jbabdi S. Rushworth M. F. S. Woolrich M. W. (2007). Probabilistic diffusion tractography with multiple fibre orientations: what can we gain? Neuroimage 34, 144–155. 10.1016/j.neuroimage.2006.09.018 17070705 PMC7116582

[B5] Behrens T. E. J. Sporns O. (2012). Human connectomics. Curr. Opin. Neurobiol. 22, 144–153. 10.1016/j.conb.2011.08.005 21908183 PMC3294015

[B6] Bota M. Talpalaru S. Hintiryan H. Dong H.-W. Swanson L. W. (2014). Bams2 workspace: a comprehensive and versatile neuroinformatic platform for collating and processing neuroanatomical connections. J. Comp. Neurol. 522, 3160–3176. 10.1002/cne.23592 24668342 PMC4107155

[B7] Caspers S. Eickhoff S. B. Zilles K. Amunts K. (2013). Microstructural grey matter parcellation and its relevance for connectome analyses. Neuroimage 80, 18–26. 10.1016/j.neuroimage.2013.04.003 23571419 PMC8010271

[B8] Craddock R. C. Jbabdi S. Yan C.-G. Vogelstein J. T. Castellanos F. X. Martino A. D.. (2013). Imaging human connectomes at the macroscale. Nat. Methods 10, 524–539. 10.1038/nmeth.2482 23722212 PMC4096321

[B9] Daducci A. Gerhard S. Griffa A. Lemkaddem A. Cammoun L. Gigandet X.. (2012). The connectome mapper: an open-source processing pipeline to map connectomes with mri. PLoS ONE 7:e48121. 10.1371/journal.pone.0048121 23272041 PMC3525592

[B10] Dale A. M. Fischl B. Sereno M. I. (1999). Cortical surface-based analysis. i. segmentation and surface reconstruction. Neuroimage 9, 179–194. 10.1006/nimg.1998.0395 9931268

[B11] Dameron O. Rubin D. L. Musen M. A. (2005). Challenges in converting frame-based ontology into owl: the foundational model of anatomy case-study. AMIA Annu. Symp. Proc. 181–185. 16779026 PMC1560487

[B12] Desikan R. S. Segonne F. Fischl B. Quinn B. T. Dickerson B. C. Blacker D.. (2006). An automated labeling system for subdividing the human cerebral cortex on MRI scans into gyral based regions of interest. Neuroimage 31, 968–980. 10.1016/j.neuroimage.2006.01.021 16530430

[B13] Detwiler L. T. Suciu D. Franklin J. D. Moore E. B. Poliakov A. V. Lee E. S.. (2009). Distributed xquery-based integration and visualization of multimodality brain mapping data. Front. Neuroinform. 3:2. 10.3389/neuro.11.002.2009 19198662 PMC2636687

[B14] Essen D. C. V. Ugurbil K. (2012). The future of the human connectome. Neuroimage 62, 1299–1310. 10.1016/j.neuroimage.2012.01.032 22245355 PMC3350760

[B15] Fischl B. van der Kouwe A. Destrieux C. Halgren E. Sgonne F. Salat D. H.. (2004). Automatically parcellating the human cerebral cortex. Cereb. Cortex 14, 11–22. 10.1093/cercor/bhg087 14654453

[B16] Fortino A. Zalesky A. Breakspear M. (2013). Graph analysis of the human connectome: promise, progress, and pitfalls. Neuroimage 80, 426–444. 10.1016/j.neuroimage.2013.04.087 23643999

[B17] Golbreich C. Grosjean J. Darmoni S. J. (2013). The foundational model of anatomy in owl 2 and its use. Artif. Intell. Med. 57, 119–132. 10.1016/j.artmed.2012.11.002 23273493

[B18] Golbreich C. Zhang S. Bodenreider O. (2006). The foundational model of anatomy in owl: experience and perspectives. Web Semant. 4, 181–195. 10.1016/j.websem.2006.05.007 18360535 PMC2270940

[B19] Gruber T. R. (1995). Toward principles for the design of ontologies used for knowledge sharing? Int. J. Hum. Comput. Stud. 43, 907–928. 11417202

[B20] Hagmann P. Cammoun L. Gigandet X. Meuli R. Honey C. J. Wedeen V. J.. (2008). Mapping the structural core of human cerebral cortex. PLoS Biol. 6:e159. 10.1371/journal.pbio.0060159 18597554 PMC2443193

[B21] Hagmann P. Kurant M. Gigandet X. Thiran P. Wedeen V. J. Meuli R.. (2007). Mapping human whole-brain structural networks with diffusion mri. PLoS ONE 2:e597. 10.1371/journal.pone.0000597 17611629 PMC1895920

[B22] Honey C. J. Thivierge J.-P. Sporns O. (2010). Can structure predict function in the human brain? Neuroimage 52, 766–776. 10.1016/j.neuroimage.2010.01.071 20116438

[B23] Hua K. Zhang J. Wakana S. Jiang H. Li X. Reich D. S.. (2008). Tract probability maps in stereotaxic spaces: analyses of white matter anatomy and tract-specific quantification. Neuroimage 39, 336–347. 10.1016/j.neuroimage.2007.07.053 17931890 PMC2724595

[B24] Jbabdi S. Johansen-Berg H. (2011). Tractography: where do we go from here? Brain Conn. 1, 169–183. 10.1089/brain.2011.0033 22433046 PMC3677805

[B25] Jbabdi S. Woolrich M. W. Behrens T. E. J. (2009). Multiple-subjects connectivity-based parcellation using hierarchical dirichlet process mixture models. Neuroimage 44, 373–384. 10.1016/j.neuroimage.2008.08.044 18845262

[B26] Johansen-Berg H. Behrens T. E. J. Robson M. D. Drobnjak I. Rushworth M. F. S. Brady J. M.. (2004). Changes in connectivity profiles define functionally distinct regions in human medial frontal cortex. Proc. Natl. Acad. Sci. U.S.A. 101, 13335–13340. 10.1073/pnas.0403743101 15340158 PMC516567

[B27] Jones D. K. Knösche T. R. Turner R. (2013). White matter integrity, fiber count, and other fallacies: the do's and don'ts of diffusion mri. Neuroimage 73, 239–254. 10.1016/j.neuroimage.2012.06.081 22846632

[B28] Larson S. D. Martone M. E. (2013). Neurolex.org: an online framework for neuroscience knowledge. Front. Neuroinform. 7:18. 10.3389/fninf.2013.00018 24009581 PMC3757470

[B29] Leergaard T. B. Hilgetag C. C. Sporns O. (2012). Mapping the connectome: multi-level analysis of brain connectivity. Front. Neuroinform. 6:14. 10.3389/fninf.2012.00014 22557964 PMC3340894

[B30] Mechouche A. Morandi X. Golbreich C. Gibaud B. (2009). A hybrid system using symbolic and numeric knowledge for the semantic annotation of sulco-gyral anatomy in brain mri images. IEEE Trans. Med. Imaging 28, 1165–1178. 10.1109/TMI.2009.2026746 19622437

[B31] Mori S. Wakana S. van Zijl P. C. M. Nagae-Poetscher L. M. (2005). MRI Atlas of Human White Matter (Amsterdam: Elsevier).

[B32] Neuhaus F. Vizedom A. (2013). Towards ontology evaluation across the life cycle: The Ontology Summit 2013. Appl. Ontol. 8, 179–194 10.3233/AO-130125

[B33] Nichols B. N. Mejino J. L. Detwiler L. T. Nilsen T. T. Martone M. E. Turner J. A.. (2014). Neuroanatomical domain of the foundational model of anatomy ontology. J. Biomed. Semant. 5:1. 10.1186/2041-1480-5-1 24398054 PMC3944952

[B34] Noy N. F. Rubin D. L. (2008). Translating the foundational model of anatomy into owl. Web Semant. 6, 133–136. 10.1016/j.websem.2007.12.001 18688289 PMC2500209

[B35] Poupon C. Poupon F. Allirol L. Mangin J.-F. (2006). A database dedicated to anatomo-functional study of human brain connectivity, in Twelfth Annual Meeting of the Organization for Human Brain Mapping (HBM) (Florence), 1–8.

[B36] Reese T. G. Heid O. Weisskoff R. M. Wedeen V. J. (2003). Reduction of eddy-current-induced distortion in diffusion MRI using a twice-refocused spin echo. Magn. Reson. Med. 49, 177–182. 10.1002/mrm.10308 12509835

[B37] Rosse C. Mejino J. L. V. (2003). A reference ontology for biomedical informatics: the foundational model of anatomy. J. Biomed. Inform. 36, 478–500. 10.1016/j.jbi.2003.11.007 14759820

[B38] Rubin D. L. Noy N. F. Musen M. A. (2007). Protg: a tool for managing and using terminology in radiology applications. J. Digit. Imaging 20(Suppl. 1), 34–46. 10.1007/s10278-007-9065-0 17687607 PMC2039856

[B39] Russ T. A. Ramakrishnan C. Hovy E. H. Bota M. Burns G. A. P. C. (2011). Knowledge engineering tools for reasoning with scientific observations and interpretations: a neural connectivity use case. BMC Bioinform. 12:351. 10.1186/1471-2105-12-351 21859449 PMC3176268

[B40] Shaw M. Detwiler L. T. Noy N. Brinkley J. Suciu D. (2011). vsparql: a view definition language for the semantic web. J. Biomed. Inform. 44, 102–117. 10.1016/j.jbi.2010.08.008 20800106 PMC3042057

[B41] Smith B. Ceusters W. Klagges B. Khler J. Kumar A. Lomax J.. (2005). Relations in biomedical ontologies. Genome Biol. 6:R46. 10.1186/gb-2005-6-5-r46 15892874 PMC1175958

[B42] Smith S. M. Miller K. L. Salimi-Khorshidi G. Webster M. Beckmann C. F. Nichols T. E.. (2011). Network modelling methods for fmri. Neuroimage 54, 875–891. 10.1016/j.neuroimage.2010.08.063 20817103

[B43] Sporns O. (2013). The human connectome: origins and challenges. Neuroimage 80, 53–61. 10.1016/j.neuroimage.2013.03.023 23528922

[B44] Sporns O. Tononi G. Ktter R. (2005). The human connectome: a structural description of the human brain. PLoS Comput. Biol. 1:e42. 10.1371/journal.pcbi.0010042 16201007 PMC1239902

[B45] Swanson L. W. Bota M. (2010). Foundational model of structural connectivity in the nervous system with a schema for wiring diagrams, connectome, and basic plan architecture. Proc. Natl. Acad. Sci. U.S.A. 107, 20610–20617. 10.1073/pnas.1015128107 21078980 PMC2996420

[B46] Talairach J. Tournoux P. (1988). Co-Planar Stereotaxic Atlas of the Human Brain. New York, NY: Thieme medical publishers.

[B47] Turner J. A. Mejino J. L. V. Brinkley J. F. Detwiler L. T. Lee H. J. Martone M. E.. (2010). Application of neuroanatomical ontologies for neuroimaging data annotation. Front. Neuroinform. 4:10. 10.3389/fninf.2010.00010 20725521 PMC2912099

[B48] Tzourio-Mazoyer N. Landeau B. Papathanassiou D. Crivello F. Etard O. Delcroix N.. (2002). Automated anatomical labeling of activations in spm using a macroscopic anatomical parcellation of the mni mri single-subject brain. Neuroimage 15, 273–289. 10.1006/nimg.2001.0978 11771995

[B49] Wakana S. Caprihan A. Panzenboeck M. M. Fallon J. H. Perry M. Gollub R. L.. (2007). Reproducibility of quantitative tractography methods applied to cerebral white matter. Neuroimage 36, 630–644. 10.1016/j.neuroimage.2007.02.049 17481925 PMC2350213

[B50] Wassermann D. Makris N. Rathi Y. Shenton M. Kikinis R. Kubicki M.. (2013). On describing human white matter anatomy: the white matter query language. Med. Image Comput. Comput. Assist. Interv. 16(Pt 1), 647–654. 10.1007/978-3-642-40811-3/81 24505722 PMC4029160

[B51] Yendiki A. Panneck P. Srinivasan P. Stevens A. Zllei L. Augustinack J.. (2011). Automated probabilistic reconstruction of white-matter pathways in health and disease using an atlas of the underlying anatomy. Front. Neuroinform. 5:23. 10.3389/fninf.2011.00023 22016733 PMC3193073

[B52] Zalesky A. Fornito A. Seal M. L. Cocchi L. Westin C.-F. Bullmore E. T.. (2011). Disrupted axonal fiber connectivity in schizophrenia. Biol. Psychiatry 69, 80–89. 10.1016/j.biopsych.2010.08.022 21035793 PMC4881385

[B53] Zilles K. Amunts K. (2010). Centenary of brodmann's map–conception and fate. Nat. Rev. Neurosci. 11, 139–145. 10.1038/nrn2776 20046193

